# Whole-genome characterization of neonatal group B Streptococcus infection isolates and contemporaneous maternal colonizing isolates in Southwest China

**DOI:** 10.3389/fmicb.2026.1871541

**Published:** 2026-07-08

**Authors:** Ziyi Yan, Liyuan Mu, Chenglin Miao, Yunhan Fu, Li Liu, Xingxin Liu, Jingjing Luo, Wenjing Wu, Jiaji Ling, Liting Liang, Yali Cui, Yongmei Jiang, Linghan Kuang

**Affiliations:** 1 Department of Laboratory Medicine, West China Second University Hospital, Sichuan University, Chengdu, China; 2 Key Laboratory of Birth Defects and Related Diseases of Women and Children (Sichuan University), Ministry of Education, Chengdu, China; 3 West China School of Medicine, Sichuan University, Chengdu, China; 4 Department of Blood Transfusion, Beijing Anzhen Nanchong Hospital of Capital Medical University and Nanchong Central Hospital, Nanchong, China; 5 Department of Laboratory Medicine, West China Second University Hospital (Tianfu), Sichuan University (Sichuan Provincial Children's Hospital), Meishan, China; 6 Department of Laboratory Medicine, Meishan Women and Children's Hospital, Meishan, China; 7 Department of Laboratory Medicine, Chengdu Hi-Tech Zone Hospital for Women and Children (Chengdu Hi-Tech Zone Hospital for Maternal and Child Healthcare), Chengdu, China

**Keywords:** antimicrobial resistance, clonal complex, group B Streptococcus, maternal colonization, neonatal infection, *Streptococcus agalactiae*, virulence genes, whole-genome sequencing

## Abstract

**Background:**

Regional genomic data on neonatal GBS infection in Southwest China remain limited, particularly for studies integrating neonatal infection isolates with contemporaneous maternal colonizing isolates. This study defined the antimicrobial susceptibility, molecular composition, resistance and virulence gene profiles, and phylogenetic context of neonatal GBS infection in this region.

**Methods:**

We retrospectively analyzed 52 neonatal GBS infection isolates collected from March 2020 to March 2025 and compared them with 52 maternal vaginal colonizing isolates selected from the same period. Antimicrobial susceptibility testing, whole-genome sequencing, capsular serotyping, multilocus sequence typing, clonal complex assignment, gene screening, and core-genome phylogenetic analysis were performed. The 52 neonatal infection isolates were further contextualized with 1,060 publicly available blood- or cerebrospinal fluid-derived genomes from PubMLST.

**Results:**

All neonatal infection isolates were susceptible to penicillin and ampicillin, whereas resistance to erythromycin, clindamycin, tetracycline, and moxifloxacin was common. Serotype III was the most frequent serotype, followed by Ib. Compared with maternal colonizing isolates, neonatal infection isolates showed significant enrichment of ST17/CC17 and the III-2/CC17 lineage. The predominant neonatal infection-associated lineages were Ib/CC12, III-2/CC17, and III-1/CC19. Resistance and virulence gene carriage was strongly structured by clonal background, with several aminoglycoside resistance genes and *fbsB* concentrated in CC17. Global phylogenetic analysis showed that local neonatal infection isolates were embedded within established GBS lineages.

**Conclusion:**

Neonatal GBS infection in Southwest China was associated with selected phylogenetic backgrounds rather than directly mirroring the maternal colonizing population. These findings support integrated surveillance of antimicrobial susceptibility, molecular lineages, virulence markers, and vaccine-relevant serotypes.

## 1 Introduction

*Streptococcus agalactiae* (*S. agalactiae*), clinically known as group B Streptococcus (GBS), is a Gram-positive streptococcal species defined by the Lancefield group B carbohydrate antigen, a peptidoglycan-anchored cell-wall polysaccharide that underlies the conventional “group B” designation (Caliot et al., 2012). In clinical microbiology and perinatal medicine, the term GBS is therefore used to refer to *S. agalactiae*, and this organism is recognized as a major pathogen in pregnancy, fetal infection, and neonatal disease (Manuel et al., 2024; Raabe and Shane, 2019). GBS commonly colonizes the maternal gastrointestinal and genitourinary tracts, with an estimated 19.7 million pregnant women worldwide carrying rectovaginal GBS in 2020 (Gonçalves et al., 2022). Maternal colonization provides the principal reservoir for fetal and neonatal exposure, and transmission may occur in utero or during vaginal delivery, leading to adverse outcomes including preterm birth, stillbirth, early-onset neonatal disease, sepsis, pneumonia, and meningitis (Manuel et al., 2024; Gonçalves et al., 2022). Despite preventive strategies based on maternal screening and intrapartum antibiotic prophylaxis, GBS remains an important cause of neonatal morbidity and mortality, and global burden estimates continue to highlight substantial disease in early life, particularly through sepsis, meningitis, and long-term neurodevelopmental sequelae among survivors (Gonçalves et al., 2022).

Because GBS is the clinical designation of *S. agalactiae*, we use “GBS” when referring to clinical disease, clinical isolates, maternal colonization, and surveillance-relevant contexts, while retaining *S. agalactiae* for formal taxonomic, database, and species-level genomic descriptions. Current clinical management is mainly based on antenatal screening for maternal colonization, intrapartum antibiotic prophylaxis to reduce early-onset neonatal infection, and β-lactam-based treatment once neonatal GBS infection is suspected or confirmed (Schrag et al., 2002; Van Dyke et al., 2009; Le Doare et al., 2017; Panneflek et al., 2024). However, these clinical strategies do not fully capture the bacterial heterogeneity underlying maternal colonization and neonatal disease. At the population level, *S. agalactiae* is structured by capsular serotype and genetic lineage, with infant disease concentrated in a limited number of capsular backgrounds, particularly serotypes Ia, Ib, II, III, and V (Madrid et al., 2017). Genome-based studies have further shown that human-associated *S. agalactiae* is organized into successful clonal backgrounds, including ST17/CC17, CC19, CC23, and other regionally variable lineages, and that these backgrounds may differ in antimicrobial resistance, virulence-associated gene content, and host adaptation (Da Cunha et al., 2014; Chaguza et al., 2022; Gori et al., 2020). Because serotype composition, clonal structure, and resistance or virulence marker profiles may vary across geographic regions and over time, regional genomic studies are needed rather than relying solely on global or national aggregate data. This is particularly important in settings where neonatal infection isolates and contemporaneous maternal colonizing isolates have not been analyzed together, limiting the ability to define the local bacterial backgrounds associated with neonatal GBS infection.

Despite increasing genomic data on *S. agalactiae*, region-specific information from Southwest China remains limited, particularly for studies that evaluate neonatal GBS infection isolates together with contemporaneous maternal GBS colonizing isolates. To address this gap, we conducted a whole-genome sequencing-based study of neonatal GBS infection isolates collected from a major women's and children's medical center in Southwest China and compared them with maternal vaginal colonizing isolates selected from the same study period. We characterized antimicrobial susceptibility, capsular serotype, ST/CC distribution, resistance- and virulence-related gene carriage, and core-genome phylogenetic structure. To further contextualize the local neonatal infection isolates, we also compared them with publicly available blood/CSF-derived *S. agalactiae* genomes from the PubMLST database. This study aimed to define the regional molecular composition and clinically relevant genomic features of neonatal GBS infection in Southwest China, thereby providing baseline data for clinical management, maternal prevention, and vaccine-oriented surveillance.

## 2 Materials and methods

### 2.1 Patients and isolates

We retrospectively included 52 nonduplicate neonatal clinical GBS infection isolates collected from March 2020 to March 2025 at West China Second University Hospital/National Regional Medical Center (Southwest China), one of the largest specialized hospitals for women and children in China. To provide contemporaneous population-level comparators, 52 maternal vaginal GBS colonizing isolates were selected from the same study period according to the criteria described below, resulting in a final local study collection of 104 isolates. The hospital's clinical laboratory has been accredited by the College of American Pathologists (CAP) and the ISO 15189 accreditation standard.

The inclusion criteria for patients were as follows: (1) neonates aged ≤ 28 days after birth; (2) a diagnosis of neonatal sepsis, neonatal meningitis, and/or neonatal pneumonia according to the International Classification of Diseases, 10th Revision (ICD-10); and (3) isolation, identification, and reporting of GBS from peripheral blood, cerebrospinal fluid (CSF), and/or tracheal aspirates based on standardized operating procedures (SOP) in a CAP/ISO 15189-accredited clinical microbiology laboratory.

The exclusion criteria for patients were as follows: (1) if GBS was isolated simultaneously from peripheral blood, CSF, and/or tracheal aspirates from the same neonate, only the isolate recovered from a normally sterile body fluid was retained, and duplicate isolates were excluded; and (2) if GBS was repeatedly isolated from the same neonate during the course of treatment, only the first isolate was retained, and subsequent duplicate isolates were excluded.

To compare the epidemiological characteristics of neonatal infection isolates with the contemporaneous maternal colonizing population, maternal colonizing isolates were included at a 1:1 ratio based on the final number of eligible neonatal GBS infection isolates. These maternal colonizing isolates were not obtained from the mothers of the enrolled neonates and did not represent mother–infant paired isolates. Instead, they were selected as contemporaneous population-level comparators according to predefined temporal and maternal-age matching criteria. The inclusion criteria for maternal colonizing isolates were as follows: (1) GBS isolated from the reproductive tract of pregnant women undergoing routine antenatal screening; (2) isolation within 7 days of detection of the corresponding index neonatal infection isolate; (3) maternal age within ±5 years of the mother of the corresponding index neonatal case; and (4) if multiple colonizing isolates were eligible for selection, the isolate with the closest sampling date was selected.

### 2.2 Isolate identification and antibiotic susceptibility testing

GBS isolates were collected, cultured, and identified according to standard clinical procedures as previously described (Yan et al., 2019, 2021). Peripheral blood and CSF were cultured using the BD BACTEC FX system (BD, MD, USA) and subcultured on Columbia agar with 5% sheep blood plates, whereas tracheal aspirates were directly cultured on the same medium at 35 °C for 24–48 h in 5% CO_2_. Isolates were identified by MALDI-TOF MS (Vitek MS; bioMérieux), confirmed by whole-genome signature sequence alignment with PubMLST, and then stored in 25% sterile glycerol broth at −70 °C for subsequent analysis.

Antimicrobial susceptibility testing was performed using AST panels (TDR STR-AST; Mindray, China) by the broth turbidimetric method against penicillin, ampicillin, cefepime, cefotaxime, ceftriaxone, meropenem, vancomycin, teicoplanin, daptomycin, erythromycin, clindamycin, tetracycline, moxifloxacin, linezolid, and trimethoprim–sulfamethoxazole. Quality control was performed using *Streptococcus pneumoniae* ATCC 49619 in accordance with the standards of the Clinical and Laboratory Standards Institute (CLSI). Susceptibility results were interpreted primarily according to the 36th edition of CLSI M100, and for antimicrobial agents without CLSI interpretive criteria, EUCAST (v16.0) breakpoints were applied.

### 2.3 Genome sequencing and molecular typing

Genomic DNA was extracted using a QIAamp DNA Mini Kit (QIAGEN, Germany), and libraries were prepared with an NEBNext Ultra DNA Library Prep Kit for Illumina (NEB, USA). Whole-genome sequencing was performed on the Illumina NovaSeq PE150 platform by Beijing Novogene (China). Sequencing depth was calculated as the total number of clean bases, determined using SeqKit (v2.3.0), divided by the assembled genome size for each isolate. For isolates with available clean FASTQ files, the observed sequencing depth ranged from 590.8 × to 1,141.6 × , with a median depth of 719.1 × . Reads were assembled with SPAdes (v3.14.1) and annotated with Prokka (v1.14.5) as previously described (Liu et al., 2025).

Capsular serotypes were determined from whole-genome sequencing data using the GBS-SBG database with associated scripts, which supports both short-read mapping-based and assembly-based serotype calling (Tiruvayipati et al., 2021). Sequence types were assigned using the *S. agalactiae* MLST scheme in the PubMLST database in conjunction with the mlst tool (v2.19.0) (Jolley and Maiden, 2010). Clonal complex (CC) assignments followed the PubMLST *S. agalactiae* database wherever available. For STs lacking an explicit database-defined CC, clonal relationships were inferred using the standard 6/7 shared-allele (single-locus variant) criterion of eBURST/goeBURST, with additional support from PubMed-indexed literature when necessary; otherwise, STs were considered singletons.

### 2.4 Antibiotic resistance- and virulence-related gene screening

Antimicrobial resistance gene screening was conducted using ABRicate (v1.0.1, https://github.com/tseemann/abricate) with multiple reference databases, including NCBI AMRFinderPlus (https://github.com/ncbi/amr), the Comprehensive Antibiotic Resistance Database (CARD, https://card.mcmaster.ca), and ARG-ANNOT (https://www.mediterranee-infection.com/acces-ressources/base-de-donnees/arg-annot-2), while replicon signals were screened using Pathogenwatch. Virulence gene screening was also performed using ABRicate (v1.0.1) against the Virulence Factor Database (VFDB, https://www.mgc.ac.cn/VFs/main.htm), focusing on genes associated with exotoxin-related genes, exoenzyme-related genes, adherence-related genes, and pilus island-associated genes (PI-1 and PI-2a). Hits with both sequence coverage and identity >80% were considered positive, and the corresponding isolates were classified as carriers of the respective genes.

### 2.5 Phylogenetic analysis

For comparative phylogenetic analysis, publicly available *S. agalactiae* genomes were retrieved from the PubMLST database. As of 13 March 2026, a total of 13,334 *S. agalactiae* genomes were available in PubMLST, of which 1,122 were reported to be isolated from blood or cerebrospinal fluid. These genomes were downloaded and subjected to quality control using SeqKit, Kraken2, and CheckM2. MLST and capsular serotypes were re-assessed to confirm species identity and typing consistency. After quality filtering and re-evaluation, 1,060 qualified public blood/CSF-derived genomes were retained for downstream analysis. All genome assemblies, including the 104 isolates from this study and the selected public genomes, were annotated using Prokka (v1.14.5) (Liu et al., 2025).

Core-genome phylogenies were inferred using a uniform workflow. Briefly, Roary was used to define the core genome and generate a concatenated multi-FASTA alignment of genes present in more than 99% of isolates (Page et al., 2015). To reduce redundancy before phylogenetic reconstruction, duplicate sites in the core-gene alignment were removed using SNP-sites (v2.3.3). The resulting alignment was then used for maximum-likelihood tree inference with RAxML (v8.2.10) under the GTRGAMMA nucleotide substitution model (Stamatakis, 2014). Final trees were visualized and annotated in iTOL (v6, https://itol.embl.de).

### 2.6 Statistical analysis

Statistical analyses were performed in Python (v3.13.5) using pandas (v2.2.3), NumPy (v2.3.5), SciPy (v1.17.0), and statsmodels (v0.14.6). Categorical variables were summarized as counts and percentages. Goodness-of-fit chi-square tests were used to assess whether the sex distribution and seasonal distribution of neonatal cases deviated from equal expected proportions. Comparisons of categorical variables between neonatal infection isolates and maternal colonizing isolates, including resistance- and virulence-gene carriage rates, were performed using Pearson's chi-square test or Fisher's exact test as appropriate. For comparisons involving the major clonal complexes, Pearson's chi-square test was used. The association between capsular serotype and clonal complex was evaluated using Pearson's chi-square test, and effect size was summarized by Cramér's V. For overall comparisons involving multiple serotype, ST, CC, or lineage categories, post hoc analyses were performed using one-vs.-rest 2 × 2 contingency testing, and *P*-values were adjusted for multiple comparisons using the Benjamini-Hochberg method. All tests were two-sided, and *P* < 0.05 was considered statistically significant.

## 3 Results

### 3.1 Demographic and clinical characteristics

A total of 52 neonates with GBS infection were included in this study (Table 1). There were 27 males (51.9%) and 25 females (48.1%), with no significant difference in sex distribution (χ^2^ = 0.077, *P* = 0.782). Most cases occurred within the first week of life, with 34/52 (65.4%) neonates aged 0–6 days and 18/52 (34.6%) aged 7–28 days; the median age was 0 days (P25–P75, 0–13.5 days). Regarding season of birth, 16/52 (30.8%) neonates were born in spring, 16/52 (30.8%) in summer, 8/52 (15.4%) in autumn, and 12/52 (23.1%) in winter, with no significant seasonal difference in case distribution (χ^2^ = 3.385, *P* = 0.336).

**Table 1:** Demographic and clinical characteristics of 52 neonates with GBS infection.

Characteristic	No. of patients	(%)
Sex		
Male	27	51.9
Female	25	48.1
Age (days)		
0–6	34	65.4
7–28	18	34.6
*Median age (P25–P75)*	*0 (0–13.5)*	
Season of birth		
Spring (March-May)	16	30.8
Summer (June-August)	16	30.8
Autumn (September-November)	8	15.4
Winter (December-February)	12	23.1
Diagnosis		
Neonatal sepsis	41	78.8
With meningitis	10	19.2
Neonatal pneumonia	11	21.2
Sample type		
Blood	31	59.6
Cerebrospinal fluid	10	19.2
Tracheal aspirate	11	21.2
Clinical outcome		
Improved	49	94.2
Death	1	1.9
Withdrawal of treatment	2	3.8
**Total**	**52**	**100.0**

Meningitis was recorded as a subgroup of neonatal sepsis rather than a mutually exclusive diagnostic category.

Diagnostic categories accounted for all 52 neonates. Neonatal sepsis was diagnosed in 41/52 (78.8%) neonates, including 10/52 (19.2%) with concomitant meningitis, whereas neonatal pneumonia was diagnosed in the remaining 11/52 (21.2%) neonates. Regarding clinical outcomes, 49/52 (94.2%) neonates showed clinical improvement, 1/52 (1.9%) died, and treatment was withdrawn in 2/52 (3.8%) cases.

### 3.2 Antibiotic susceptibility

**Figure 1:**
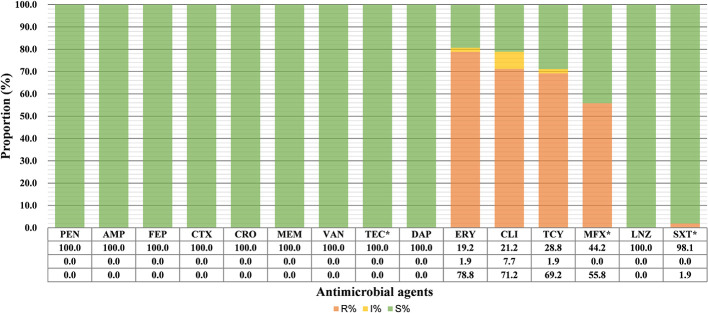
Antimicrobial susceptibility profiles of 52 neonatal infection-associated GBS isolates. Susceptible (S), intermediate (I), and resistant (R) categories were interpreted according to the 36th edition of CLSI M100 for β-hemolytic streptococci; agents marked with an asterisk (*) were interpreted according to EUCAST version 16.0. PEN, penicillin G; AMP, ampicillin; FEP, cefepime; CTX, cefotaxime; CRO, ceftriaxone; MEM, meropenem; VAN, vancomycin; TEC, teicoplanin; DAP, daptomycin; ERY, erythromycin; CLI, clindamycin; TCY, tetracycline; MFX, moxifloxacin; LNZ, linezolid; SXT, trimethoprim-sulfamethoxazole.

Antimicrobial susceptibility testing was performed for all 52 neonatal infection isolates (Figure 1). All isolates were fully susceptible to penicillin, ampicillin, cefepime, cefotaxime, ceftriaxone, meropenem, vancomycin, teicoplanin, daptomycin, and linezolid. In contrast, high resistance rates were observed for erythromycin, clindamycin, tetracycline, and moxifloxacin, with resistance detected in 78.8%, 71.2%, 69.2%, and 55.8% of isolates, respectively. Trimethoprim–sulfamethoxazole remained highly active overall, with 98.1% of isolates classified as susceptible and only 1.9% as resistant.

In this study, no statistically significant difference was identified between specimen category and resistance to the tested antimicrobial agents (erythromycin: *P* = 1.000; clindamycin: *P* = 0.681; tetracycline: *P* = 1.000; moxifloxacin: *P* = 0.507); Similarly, no statistically significant seasonal difference in resistance was observed (erythromycin: *P* = 0.454; clindamycin: *P* = 0.497; tetracycline: *P* = 0.654; moxifloxacin: *P* = 1.000).

### 3.3 Capsular serotype and ST/CC distribution

To compare the epidemiological characteristics of neonatal infection isolates and contemporaneous maternal colonizing isolates, 52 maternal colonizing isolates were additionally included according to the predefined matching criteria described in the Methods section. Therefore, the analysis in this section was based on a total of 104 GBS isolates, comprising 52 neonatal infection isolates and 52 maternal colonizing isolates.

**Figure 2:**
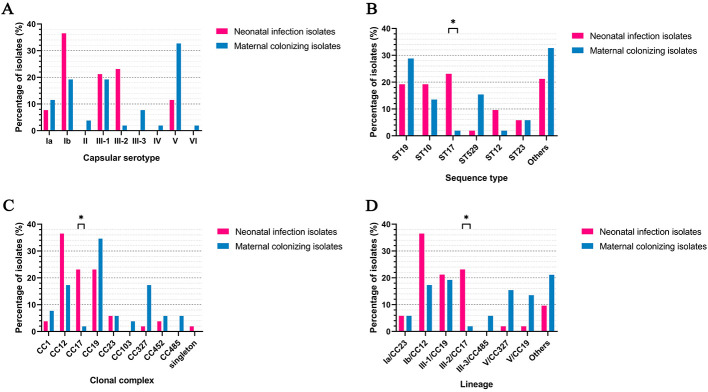
Molecular distribution of neonatal and maternal GBS isolates. A total of 104 isolates were included, comprising 52 neonatal infection-associated isolates and 52 maternal vaginal colonizing isolates. Maternal isolates were selected by systematic sampling according to maternal age and specimen submission time and were not mother–infant paired. Bars show the percentage of isolates in each group. **(A)** Capsular serotype distribution; serotype III was further separated into III-1, III-2, and III-3 to show the subtype distribution. **(B)** ST distribution. **(C)** CC distribution. **(D)** Serotype–CC lineage distribution. Each category was compared with all remaining categories using two-sided Fisher's exact tests, followed by Benjamini–Hochberg correction for multiple comparisons. Asterisks (*) indicate adjusted *P* < 0.05.

At the capsular serotype level (Figure 2A), 7 capsular serotypes were identified, namely Ia, Ib, II, III, IV, V, and VI, and the overall serotype distribution differed significantly between neonatal infection isolates and maternal colonizing isolates (χ^2^ = 14.14, *P* = 0.028). Among neonatal infection isolates, the common serotypes were III (23/52, 44.2%), Ib (19/52, 36.5%), V (6/52, 11.5%), and Ia (4/52, 7.7%). Among maternal colonizing isolates, the common serotypes were V (17/52, 32.7%), III (15/52, 28.8%), Ib (10/52, 19.2%), and Ia (6/52, 11.5%).

At the sequence type (ST) level (Figure 2B), a total of 23 STs were identified. Among them, 4 novel ST alleles were identified, namely *pheS*(202), *pheS*(203), *sdhA*(230), and *glcK*(255), all of which were detected in neonatal infection isolates. Four novel STs were also identified, namely ST2522, ST2523, ST2524, and ST2526; of these, 3 were detected in neonatal infection isolates and 1 in a maternal colonizing isolate. All newly identified alleles and STs were submitted to the PubMLST database and have been assigned official entries.

The overall ST distribution differed significantly between neonatal infection isolates and maternal colonizing isolates (χ^2^ = 40.15, *P* = 0.010). Among neonatal infection isolates, the 4 most common STs were ST17 (12/52, 23.1%), ST19 (10/52, 19.2%), ST10 (10/52, 19.2%), and ST12 (5/52, 9.6%). Among maternal colonizing isolates, the 4 most common specific STs were ST19 (15/52, 28.8%), ST529 (8/52, 15.4%), ST10 (7/52, 13.5%), and ST23 (3/52, 5.8%). Post hoc analysis showed that ST17 was significantly more frequent in neonatal infection isolates than in maternal colonizing isolates (23.1% vs. 1.9%, Benjamini-Hochberg-adjusted *P* = 0.011).

At the clonal complex (CC) level (Figure 2C), a total of 10 CC categories were identified. The overall CC distribution differed significantly between neonatal infection isolates and maternal colonizing isolates (χ^2^ = 27.35, *P* = 0.001). Among neonatal infection isolates, the 3 most common CCs were CC12 (19/52, 36.5%), CC17 (12/52, 23.1%), and CC19 (12/52, 23.1%). Among maternal colonizing isolates, the 3 most common CCs were CC19 (18/52, 34.6%), CC12 (9/52, 17.3%), and CC327 (9/52, 17.3%). Post hoc analysis showed that CC17 was significantly more frequent in neonatal infection isolates than in maternal colonizing isolates (23.1% vs. 1.9%, Benjamini-Hochberg-adjusted *P* = 0.019).

Across the 104 GBS isolates, capsular serotype was strongly associated with clonal complex (CC) (χ^2^ = 455.66, *P* < 0.001; Cramér's V = 0.740), indicating a marked non-random correspondence between serotype and genetic lineage. Specifically, Ib was predominantly associated with CC12 (28/29, 96.6%), III-1 with CC19 (21/21, 100.0%), III-2 with CC17 (13/13, 100.0%), III-3 with CC485 (3/4, 75.0%), and Ia with CC23 (6/10, 60.0%). In serotype V, two major CC backgrounds were observed, namely CC327 (9/23, 39.1%) and CC19 (8/23, 34.8%). Accordingly, the major serotype-CC combinations were classified as Ia/CC23, Ib/CC12, III-1/CC19, III-2/CC17, III-3/CC485, V/CC327, and V/CC19, with the remaining infrequent combinations grouped as Others (Figure 2D). Using this grouping strategy, the overall lineage distribution differed significantly between neonatal infection isolates and maternal colonizing isolates (χ^2^ = 28.12, *P* < 0.001). Among neonatal infection isolates, the 3 most common lineages were Ib/CC12 (19/52, 36.5%), III-2/CC17 (12/52, 23.1%), and III-1/CC19 (11/52, 21.2%). Among maternal colonizing isolates, the 3 most common lineages were III-1/CC19 (10/52, 19.2%), Ib/CC12 (9/52, 17.3%), and V/CC327 (8/52, 15.4%). Post hoc analysis showed that III-2/CC17 was significantly more frequent in neonatal infection isolates than in maternal colonizing isolates (23.1% vs. 1.9%, Benjamini-Hochberg-adjusted *P* = 0.015).

### 3.4 Antibiotic resistance-related gene analysis

A total of 21 antimicrobial resistance-related genes were detected among the 104 GBS isolates, including 6 aminoglycoside resistance genes, 8 macrolide–lincosamide–streptogramin (MLS) or macrolide–lincosamide–streptogramin B (MLSB)-associated resistance genes, 4 tetracycline resistance genes, and 3 phenicol resistance genes (Table 2). When neonatal infection isolates were compared with maternal colonizing isolates, significant differences were observed mainly in aminoglycoside resistance genes. Specifically, the carriage rates of *aadE* (32.7% vs. 1.9%; *P* < 0.001), *ant(6)-Ia* (44.2% vs. 21.2%; *P* = 0.021), and *aph(3*′*)-IIIa* (42.3% vs. 13.5%; *P* = 0.002) were significantly higher in neonatal infection isolates than in maternal colonizing isolates. No statistically significant between-group differences were identified for the other resistance genes.

**Table 2:** Carriage of antimicrobial resistance-related genes among 104 GBS isolates in Southwest China.

Resistance genes	Overall (*n =* 104)	Neonatal infection isolates (*n =* 52)	Maternal colonizing isolates (*n =* 52)	*P*-value	CC19 (*n =* 30)	CC12 (*n =* 28)	CC17 (*n =* 13)	CC327 (*n =* 10)	P-value
Aminoglycoside resistance genes									
*aadE*	18 (17.3%)	17 (32.7%)	1 (1.9%)	**< 0.001**	0 (0.0%)	7 (25.0%)	11 (84.6%)	0 (0.0%)	**< 0.001**
*ant(6)*-Ia	34 (32.7%)	23 (44.2%)	11 (21.2%)	**0.021**	11 (36.7%)	7 (25.0%)	10 (76.9%)	0 (0.0%)	**< 0.001**
*aph(2”)*-Ii	11 (10.6%)	5 (9.6%)	6 (11.5%)	1.000	8 (26.7%)	1 (3.6%)	0 (0.0%)	0 (0.0%)	**0.008**
*aph(3′)*-IIIa	29 (27.9%)	22 (42.3%)	7 (13.5%)	**0.002**	7 (23.3%)	7 (25.0%)	11 (84.6%)	0 (0.0%)	**< 0.001**
*sat4*	8 (7.7%)	4 (7.7%)	4 (7.7%)	1.000	6 (20.0%)	1 (3.6%)	0 (0.0%)	0 (0.0%)	**0.046**
*spw*	11 (10.6%)	6 (11.5%)	5 (9.6%)	1.000	4 (13.3%)	1 (3.6%)	3 (23.1%)	0 (0.0%)	0.151
Macrolide–lincosamide–streptogramin (MLS) or macrolide–lincosamide–streptogramin B (MLSB)-associated resistance genes									
*erm(A)*	8 (7.7%)	2 (3.8%)	6 (11.5%)	0.269	6 (20.0%)	1 (3.6%)	0 (0.0%)	0 (0.0%)	**0.046**
*erm(B)*	73 (70.2%)	40 (76.9%)	33 (63.5%)	0.198	15 (50.0%)	28 (100.0%)	11 (84.6%)	10 (100.0%)	**< 0.001**
*lnu(B)*	20 (19.2%)	10 (19.2%)	10 (19.2%)	1.000	12 (40.0%)	2 (7.1%)	3 (23.1%)	0 (0.0%)	**0.006**
*lnu(C)*	2 (1.9%)	0 (0.0%)	2 (3.8%)	0.495	1 (3.3%)	0 (0.0%)	0 (0.0%)	0 (0.0%)	0.632
*lsa(C)*	3 (2.9%)	2 (3.8%)	1 (1.9%)	1.000	0 (0.0%)	0 (0.0%)	0 (0.0%)	0 (0.0%)	1.000
*lsa(E)*	18 (17.3%)	9 (17.3%)	9 (17.3%)	1.000	10 (33.3%)	2 (7.1%)	3 (23.1%)	0 (0.0%)	**0.027**
*mef(A)*	17 (16.3%)	6 (11.5%)	11 (21.2%)	0.289	8 (26.7%)	2 (7.1%)	4 (30.8%)	1 (10.0%)	0.139
*msr(D)*	17 (16.3%)	6 (11.5%)	11 (21.2%)	0.289	8 (26.7%)	2 (7.1%)	4 (30.8%)	1 (10.0%)	0.139
Tetracycline resistance genes									
*tet(L)*	4 (3.8%)	3 (5.8%)	1 (1.9%)	0.618	4 (13.3%)	0 (0.0%)	0 (0.0%)	0 (0.0%)	0.067
*tet(M)*	43 (41.3%)	21 (40.4%)	22 (42.3%)	1.000	24 (80.0%)	1 (3.6%)	4 (30.8%)	0 (0.0%)	**< 0.001**
*tet(O)*	37 (35.6%)	22 (42.3%)	15 (28.8%)	0.219	8 (26.7%)	7 (25.0%)	11 (84.6%)	10 (100.0%)	**< 0.001**
*tet(S)*	1 (1.0%)	0 (0.0%)	1 (1.9%)	1.000	0 (0.0%)	0 (0.0%)	0 (0.0%)	0 (0.0%)	1.000
Phenicol resistance genes									
*cat-TC*	2 (1.9%)	1 (1.9%)	1 (1.9%)	1.000	1 (3.3%)	0 (0.0%)	0 (0.0%)	0 (0.0%)	0.632
*catA8*	3 (2.9%)	1 (1.9%)	2 (3.8%)	1.000	2 (6.7%)	0 (0.0%)	0 (0.0%)	0 (0.0%)	0.323
*catA16*	4 (3.8%)	1 (1.9%)	3 (5.8%)	0.618	4 (13.3%)	0 (0.0%)	0 (0.0%)	0 (0.0%)	0.067

Values are presented as *n* (% positive). *P*-values for neonatal infection vs. maternal colonizing isolates were calculated using Fisher's exact test. *P*-values across CC12, CC19, CC17, and CC327 were calculated using Pearson's chi-square test. Bold values indicate statistically significant *P* values (*P* < 0.05).

Given that the neonatal infection and maternal colonizing isolates showed markedly different CC distributions, we further compared resistance-gene carriage across the 4 major CCs (CC19, CC12, CC17, and CC327). Among aminoglycoside resistance genes, significant differences in carriage were observed for *aadE* (*P* < 0.001), *ant(6)-Ia* (*P* < 0.001), *aph(2”)-Ii* (*P* = 0.008), *aph(3*′*)-IIIa* (*P* < 0.001), and *sat4* (*P* = 0.046). In particular, *aadE* and *aph(3*′*)-IIIa* were highly prevalent in CC17 (both 84.6%, 11/13), whereas *aph(2”)-Ii* was mainly detected in CC19 (26.7%, 8/30). *ant(6)-Ia* was also most common in CC17 (76.9%, 10/13), followed by CC19 (36.7%, 11/30) and CC12 (25.0%, 7/28), but was not detected in CC327.

Among macrolide–lincosamide–streptogramin (MLS) or macrolide–lincosamide–streptogramin B (MLSB)-associated resistance genes, significant differences across CCs were found for *erm(A)* (*P* = 0.046), *erm(B)* (*P* < 0.001), *lnu(B)* (*P* = 0.006), and *lsa(E)* (*P* = 0.027). *erm(B)* showed particularly high carriage in CC12 and CC327 (both 100.0%), and remained frequent in CC17 (84.6%) and CC19 (50.0%). By contrast, *erm(A)* was detected mainly in CC19 (20.0%, 6/30). *lnu(B)* and *lsa(E)* were also more common in CC19 (40.0% and 33.3%, respectively) than in the other major CCs.

Among tetracycline resistance genes, significant differences across CCs were observed for *tet(M)* (*P* < 0.001) and *tet(O)* (*P* < 0.001), whereas *tet(L)* and *tet(S)* did not differ significantly. *tet(M)* was predominantly associated with CC19 (80.0%, 24/30), while *tet(O)* was highly prevalent in CC17 (84.6%, 11/13) and CC327 (100.0%, 10/10), but less common in CC19 (26.7%) and CC12 (25.0%).

**Figure 3:**
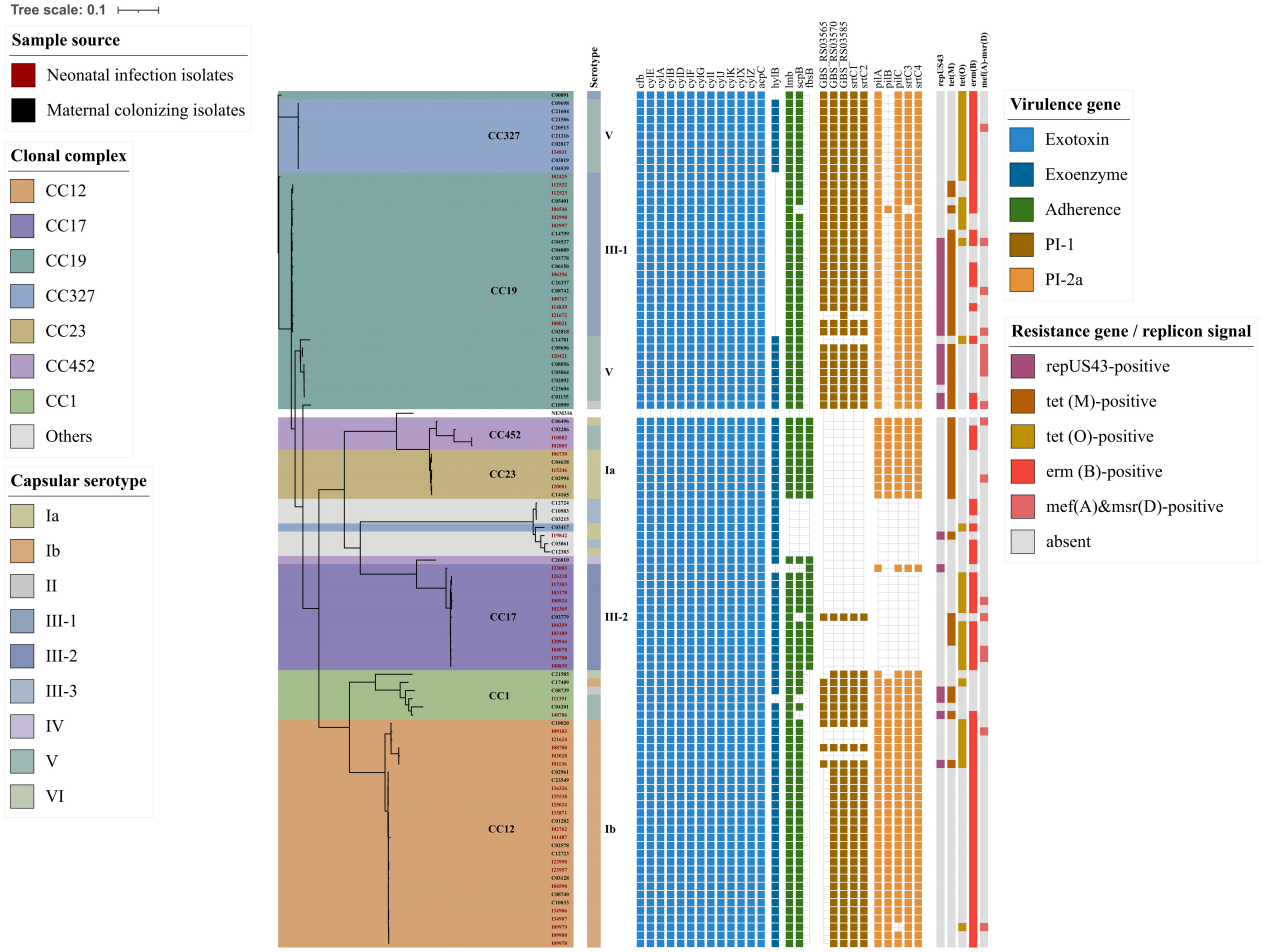
Core-genome phylogeny of 104 local GBS isolates annotated with sample source, clonal background, capsular serotype, virulence-related markers, and resistance-related markers. A total of 104 isolates were included, comprising 52 neonatal infection isolates and 52 maternal vaginal colonizing isolates. Isolate IDs in red denote neonatal infection isolates, whereas those in black denote maternal colonizing isolates. The phylogenetic tree is annotated with clonal complex (CC) and capsular serotype backgrounds, followed by presence/absence tracks for selected virulence-related genes, including exotoxin-related genes, exoenzyme-related genes, adherence-related genes, and pilus island-associated genes (PI-1 and PI-2a). Additional tracks indicate the presence or absence of selected antimicrobial resistance-related markers and replicon signal, including *mef(A)-msr(D), erm(B), tet(O), tet(M)*, and repUS43. Colored cells indicate the presence of the corresponding marker, whereas blank or gray cells indicate absence.

These lineage-associated differences in resistance-related markers were further supported by the core-genome phylogeny of the 104 isolates (Figure 3). The distribution of resistance genes on the tree was clearly non-random, with *tet(M)*-, *tet(O)*-, and *erm(B)*-positive isolates showing evident clustering rather than a scattered pattern. Likewise, the replicon signal repUS43 was detected in a restricted subset of isolates and tended to co-occur with particular resistance-gene backgrounds, rather than being evenly distributed across the population. This pattern suggests that the carriage of several resistance determinants, as well as repUS43, was linked to specific phylogenetic backgrounds and may reflect the circulation of distinct resistance-associated genetic elements in this collection.

Overall, the most frequently detected resistance genes among the 104 isolates were *erm(B)* (70.2%, 73/104), *tet(M)* (41.3%, 43/104), *tet(O)* (35.6%, 37/104), *ant(6)-Ia* (32.7%, 34/104), and *aph(3*′*)-IIIa* (27.9%, 29/104). In contrast, the remaining genes were detected at relatively low frequencies, including *aadE* and *lsa(E)* (both 17.3%), *mef(A)* and *msr(D)* (both 16.3%), *aph(2”)-Ii* and *spw* (both 10.6%), *erm(A)* and *sat4* (both 7.7%), *tet(L)* and *catA16* (both 3.8%), *lsa(C)* and *catA8* (both 2.9%), and *lnu(C), tet(S)*, and *cat-TC* ( ≤ 1.9%). These findings suggest that the observed differences in resistance-gene carriage between neonatal infection isolates and maternal colonizing isolates were largely associated with differences in lineage composition, particularly the distribution of major CCs.

### 3.5 Virulence-related gene analysis

A total of 27 virulence-related genes or gene clusters were analyzed among the 104 GBS isolates (Table 3 and Figure 3). Notably, all isolates carried the exotoxin-related gene *cfb* and the *cyl* operon genes (*cylA, cylB, cylD, cylE, cylF, cylG, cylI, cylJ, cylK, cylX*, and *cylZ*), indicating that these hemolysin-associated virulence determinants were highly conserved in both neonatal infection isolates and maternal colonizing isolates.

**Table 3:** Carriage of virulence-related genes among 104 GBS isolates in Southwest China.

Virulence genes	Overall (*n =* 104)	Neonatal infection isolates (*n =* 52)	Maternal colonizing isolates (*n =* 52)	*P*-value	CC19 (*n =* 30)	CC12 (*n =* 28)	CC17 (*n =* 13)	CC327 (*n =* 10)	*P*-value
Exotoxin-related genes									
*cfb*	104 (100.0%)	52 (100.0%)	52 (100.0%)	1.000	30 (100.0%)	28 (100.0%)	13 (100.0%)	10 (100.0%)	1.000
*cyl* operon genes (*cylA, cylB, cylD, cylE, cylF, cylG, cylI, cylJ, cylK, cylX, cylZ*)	104 (100.0%)	52 (100.0%)	52 (100.0%)	1.000	30 (100.0%)	28 (100.0%)	13 (100.0%)	10 (100.0%)	1.000
Exoenzyme-related genes									
*acpC*	104 (100.0%)	52 (100.0%)	52 (100.0%)	1.000	30 (100.0%)	28 (100.0%)	13 (100.0%)	10 (100.0%)	1.000
*hylB*	81 (77.9%)	39 (75.0%)	42 (80.8%)	0.637	9 (30.0%)	28 (100.0%)	12 (92.3%)	10 (100.0%)	**< 0.001**
Adherence-related genes									
*lmb*	96 (92.3%)	50 (96.2%)	46 (88.5%)	0.269	30 (100.0%)	28 (100.0%)	12 (92.3%)	9 (90.0%)	0.151
*scpB*	92 (88.5%)	47 (90.4%)	45 (86.5%)	0.760	29 (96.7%)	28 (100.0%)	11 (84.6%)	9 (90.0%)	0.159
*fbsB*	24 (23.1%)	17 (32.7%)	7 (13.5%)	**0.035**	0 (0.0%)	0 (0.0%)	13 (100.0%)	0 (0.0%)	**< 0.001**
PI-1-related genes									
GBS_RS03565	46 (44.2%)	16 (30.8%)	30 (57.7%)	**0.010**	28 (93.3%)	3 (10.7%)	1 (7.7%)	9 (90.0%)	**< 0.001**
GBS_RS03570	69 (66.3%)	30 (57.7%)	39 (75.0%)	0.096	28 (93.3%)	25 (89.3%)	1 (7.7%)	9 (90.0%)	**< 0.001**
GBS_RS03585	70 (67.3%)	31 (59.6%)	39 (75.0%)	0.143	29 (96.7%)	25 (89.3%)	1 (7.7%)	9 (90.0%)	**< 0.001**
*srtC1*	69 (66.3%)	30 (57.7%)	39 (75.0%)	0.096	28 (93.3%)	25 (89.3%)	1 (7.7%)	9 (90.0%)	**< 0.001**
*srtC2*	69 (66.3%)	30 (57.7%)	39 (75.0%)	0.096	28 (93.3%)	25 (89.3%)	1 (7.7%)	9 (90.0%)	**< 0.001**
PI-2a-related genes									
*pilA*	84 (80.8%)	40 (76.9%)	44 (84.6%)	0.456	30 (100.0%)	28 (100.0%)	1 (7.7%)	9 (90.0%)	**< 0.001**
*pilB*	44 (42.3%)	27 (51.9%)	17 (32.7%)	0.074	1 (3.3%)	28 (100.0%)	0 (0.0%)	0 (0.0%)	**< 0.001**
*pilC*	83 (79.8%)	39 (75.0%)	44 (84.6%)	0.329	30 (100.0%)	27 (96.4%)	1 (7.7%)	9 (90.0%)	**< 0.001**
*srtC3*	83 (79.8%)	39 (75.0%)	44 (84.6%)	0.329	29 (96.7%)	28 (100.0%)	1 (7.7%)	9 (90.0%)	**< 0.001**
*srtC4*	84 (80.8%)	40 (76.9%)	44 (84.6%)	0.456	30 (100.0%)	28 (100.0%)	1 (7.7%)	9 (90.0%)	**< 0.001**

Values are presented as *n* (% positive). *P*-values for neonatal infection vs. maternal colonizing isolates were calculated using Fisher's exact test. *P*-values across CC12, CC19, CC17, and CC327 were calculated using Pearson's chi-square test. Bold values indicate statistically significant *P* values (*P* < 0.05).

Among the non-universal virulence genes, the most prominent difference between neonatal infection isolates and maternal colonizing isolates was observed for the adherence-related gene *fbsB*, which was detected significantly more frequently in neonatal infection isolates than in maternal colonizing isolates (32.7% vs. 13.5%; *P* = 0.035). In contrast, no significant between-group differences were identified for the other selected exoenzyme- or adherence-related genes, including *hylB, lmb*, and *scpB*.

Given the marked differences in CC distribution between neonatal infection isolates and maternal colonizing isolates, we further compared virulence-gene carriage across the four major CCs (CC19, CC12, CC17, and CC327). Among exoenzyme-related genes, *hylB* showed significant variation across CCs (*P* < 0.001), with high carriage in CC12 (100.0%), CC17 (92.3%), and CC327 (100.0%), but substantially lower carriage in CC19 (30.0%). Among adherence-related genes, *fbsB* also differed significantly across CCs (*P* < 0.001) and showed a striking lineage restriction, being detected exclusively in CC17 and absent from CC19, CC12, and CC327. By contrast, *lmb* and *scpB* were broadly distributed and did not differ significantly among the four major CCs.

Pilus island-related genes also showed distinct CC-associated carriage patterns. For PI-1-related genes, GBS_RS03565 was significantly more common in maternal colonizing isolates than in neonatal infection isolates (57.7% vs. 30.8%; *P* = 0.010), whereas the other PI-1-related genes did not differ significantly between the two groups. However, all 5 PI-1-related genes displayed significant heterogeneity across CCs (all *P* < 0.001). In particular, GBS_RS03565 was highly prevalent in CC19 (93.3%) and CC327 (90.0%), but uncommon in CC12 (10.7%) and CC17 (7.7%). Similarly, GBS_RS03570, GBS_RS03585, *srtC1*, and *srtC2* were frequent in CC19, CC12, and CC327, but rare in CC17.

For PI-2a-related genes, no significant differences were observed between neonatal infection isolates and maternal colonizing isolates. Nevertheless, all 5 PI-2a-related genes (*pilA, pilB, pilC, srtC3*, and *srtC4*) differed significantly across CCs (all *P* < 0.001). *pilA, pilC, srtC3*, and *srtC4* were highly prevalent in CC19, CC12, and CC327, but were rarely detected in CC17. In contrast, *pilB* showed a distinct pattern, being strongly associated with CC12, but rarely detected in CC19 (3.3%) and absent from CC17 and CC327.

Overall, the phylogenetic annotation in Figure 3 further supported that virulence-gene carriage was highly structured by genetic background. While core exotoxin-related determinants were universally conserved, several exoenzyme-, adherence-, and pilus island-related genes showed clear lineage-associated clustering, particularly *fbsB* in CC17, reduced *hylB* carriage in CC19, and the contrasting PI-1/PI-2a gene patterns across the major clonal complexes.

### 3.6 Global phylogenetic context of local neonatal infection isolates

**Figure 4:**
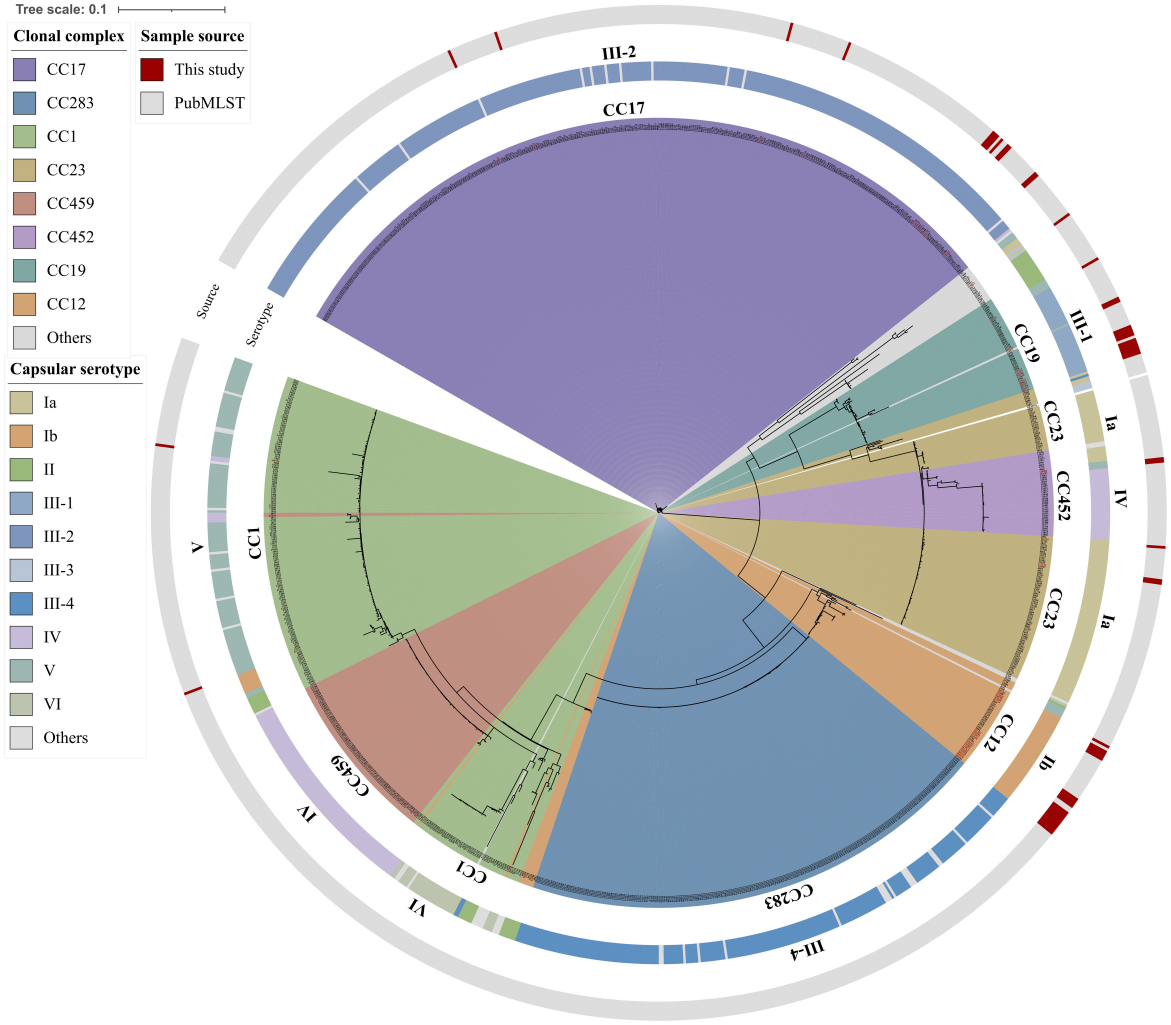
Global phylogenetic context of 52 local neonatal GBS infection isolates within 1,060 publicly available *S. agalactiae* genomes recovered from blood or cerebrospinal fluid from the PubMLST database. A total of 1,112 isolates were included, comprising 52 neonatal infection isolates from this study and 1,060 publicly available isolates recovered from blood or cerebrospinal fluid. The phylogenetic tree was constructed based on core-genome sequences. From inner to outer rings, annotations indicate clonal complex (CC), capsular serotype, and sample source. Isolates from this study are marked in red, whereas PubMLST isolates are marked in gray.

To place the 52 local neonatal infection isolates into a broader genomic background, 1,060 publicly available *S. agalactiae* genomes recovered from blood or cerebrospinal fluid were retrieved from the PubMLST database and analyzed together with the 52 isolates from this study, generating a core-genome phylogeny of 1,112 isolates in total (Figure 4). At the overall phylogenetic level, the tree was structured by major clonal complex (CC) and capsular serotype backgrounds. The 52 local neonatal infection isolates were embedded within several globally represented lineages rather than forming a single independent branch. However, their placement was not evenly dispersed across the tree. Instead, local isolates showed visible aggregation within selected lineages, particularly within the CC19/III-1 and CC12/Ib clades, with additional representation in the CC17/III-2 background, whereas only sporadic local isolates were observed in other major clades. These findings indicate that the neonatal infection isolates from Southwest China share major phylogenetic backgrounds with globally circulating *S. agalactiae* populations represented by public blood/CSF-derived genomes, while also showing non-random local clustering within specific CC-associated lineages.

## 4 Discussion

Antimicrobial susceptibility is a clinically important starting point for discussing neonatal GBS infection because it directly relates to both perinatal prevention and postnatal treatment. Current prevention strategies are centered on antenatal screening for maternal colonization and intrapartum antibiotic prophylaxis for women who are colonized or otherwise considered at increased risk of transmitting GBS to their newborns (Manuel et al., 2024). Penicillin remains the preferred agent for intrapartum prophylaxis, with ampicillin as an acceptable alternative, whereas clindamycin is reserved for selected women with significant β-lactam allergy only when *in vitro* susceptibility has been confirmed (Dhudasia et al., 2021). After birth, empiric therapy for suspected early-onset neonatal GBS infection commonly includes ampicillin plus an aminoglycoside, whereas definitive therapy after microbiological confirmation continues to rely mainly on penicillin or ampicillin (Manuel et al., 2024; Coggins and Puopolo, 2024).

Against this clinical background, the susceptibility profile observed in the 52 neonatal infection isolates from Southwest China is reassuring in that all isolates remained susceptible to penicillin, ampicillin, cefepime, cefotaxime, ceftriaxone, meropenem, vancomycin, teicoplanin, daptomycin, and linezolid, supporting the continued suitability of β-lactams as the backbone of prophylaxis and targeted treatment in this setting. However, the high resistance rates to erythromycin, clindamycin, and tetracycline, together with the substantial proportion of moxifloxacin-resistant isolates, indicate that alternative agents should not be assumed to retain reliable activity.

This pattern is broadly concordant with previous Chinese data showing preserved β-lactam activity but high non-β-lactam resistance in neonatal GBS infection. In an earlier Chinese series of invasive neonatal isolates in 2015, all strains were susceptible to penicillin, whereas resistance to erythromycin, clindamycin, and tetracycline reached 92.5%, 87.5%, and 100.0%, respectively, all higher than the corresponding rates among the 52 neonatal infection isolates from Southwest China (78.8%, 71.2%, and 69.2%) (Wang et al., 2015). In contrast, erythromycin and clindamycin resistance rates in the present study were close to those reported in a later multicenter neonatal study from China in 2019, in which 75.9% of isolates were resistant to erythromycin and 77.3% to clindamycin, while all GBS isolates remained susceptible to penicillin and ampicillin (Tan et al., 2021). Taken together, these comparisons support the continued use of penicillin-based prophylaxis and treatment in this setting.

Globally, serotype III has been the dominant capsular type in infant invasive GBS disease, accounting for 61.5% of cases, and 97.0% of invasive infant isolates belong to serotypes Ia, Ib, II, III, and V (Madrid et al., 2017). In the present neonatal infection cohort, serotype III remained the most common serotype, but at a lower proportion (44.2%), whereas serotype Ib accounted for 36.5%, markedly higher than the global proportion of 5.7% reported in that meta-analysis. A similar East Asian pattern has also been reported in Taiwan, where serotype III caused 53.9% of invasive infant episodes, followed by Ia (17.0%) and Ib (10.4%) (Lo et al., 2017). Taken together, these comparisons suggest that serotype III remains the principal disease-associated background in Southwest China, but the contribution of serotype Ib in this study was greater than that reported in global series.

At the ST/CC level, the neonatal infection isolates showed a relatively mixed clonal structure. Although ST17 was the most common individual ST (23.1%), the overall population was led by CC12 (36.5%), followed by CC17 and CC19 (both 23.1%). This distribution was not entirely similar to earlier Chinese infant studies, in which CC17 was more prominent, accounting for 40.6% of invasive isolates in a national multicenter study (Ji et al., 2019) and 64.5% in a study from southern mainland China (Li et al., 2019). Such variation is not unexpected, given the differences in study region and collection period. The isolates analyzed in this study were collected in Southwest China during 2020–2025, whereas the previous reports did not specifically represent this region and were based on earlier cohorts.

When compared with contemporaneous maternal colonizing isolates, the neonatal infection isolate collection did not simply recapitulate the local colonizing composition. Serotype III accounted for 44.2% of neonatal infection isolates but 28.8% of maternal colonizing isolates, whereas serotype V showed the opposite pattern (11.5% vs. 32.7%). A similar compositional divergence has also been reported globally. In a meta-analysis of maternal colonizing isolates, serotype III accounted for 25% of isolates (Russell et al., 2017), whereas in a separate meta-analysis of infant invasive disease, serotype III accounted for 61.5% of cases (Madrid et al., 2017). In our dataset, this shift became more evident at the ST/CC and lineage levels: ST17 and CC17 each represented 23.1% of neonatal infection isolates but only 1.9% of colonizing isolates, and the same contrast was seen for the III-2/CC17 lineage. This pattern is compatible with earlier molecular epidemiological observations that the ST17 complex is overrepresented among invasive compared with colonizing isolates (59% vs. 38%) (Lin et al., 2006), supporting the view that neonatal GBS infection in this setting was enriched for particular phylogenetic backgrounds rather than simply reflecting the maternal colonizing reservoir. Such enrichment is biologically plausible, because ST17/CC17 has long been recognized as a hypervirulent neonatal clone (Lamy et al., 2006). In experimental studies, CC17 strains have been shown to exploit host integrins to traverse brain endothelial barriers, providing a mechanistic basis for their strong association with neonatal meningitis and invasive disease (Deshayes de Cambronne et al., 2021). More recent work has also suggested that CC17 exhibits distinctive interactions with human monocytes and macrophages compared with non-CC17 strains, further supporting the idea that this lineage possesses specific host-adaptation features relevant to invasive infection (Bourrel et al., 2024).

Among the 52 neonatal GBS infection isolates collected in Southwest China during 2020–2025, the resistance-gene profile was largely consistent with the phenotypic susceptibility results. High erythromycin and clindamycin resistance in these infection isolates was accompanied mainly by *erm(B)*, whereas tetracycline resistance was supported predominantly by *tet(M)* and *tet(O)*. A similar pattern was reported in a recent multicenter, population-based Chinese study of invasive infant isolates, in which 77.3% carried erm genes and 83.8% carried tet cluster genes (Ji et al., 2024). Likewise, in infants from southern mainland China, *erm(B), tet(O)*, and *tet(M)* were the dominant corresponding resistance determinants, in the setting of 60.2% erythromycin resistance, 65.5% clindamycin resistance, and 93.5% tetracycline resistance (Li et al., 2019). Thus, although the absolute resistance rates among the 52 neonatal infection isolates from Southwest China were not identical to those reported previously, the underlying genotypic framework was broadly similar.

When the analysis was expanded to all 104 study isolates, including 52 neonatal infection isolates and 52 contemporaneous maternal colonizing isolates, the main between-group differences were concentrated in aminoglycoside-associated genes rather than in the major MLS- or tetracycline-resistance determinants. In this 104-isolate dataset, *aadE* and *aph(3*′*)*-IIIa were strongly concentrated in CC17, whereas *tet(M)* was mainly associated with CC19 and *tet(O)* with CC17 and CC327, suggesting that the observed resistance-gene differences were structured primarily by clonal background. This interpretation is consistent with a recent Netherlands study, in which resistance-associated genes increased over time through the clonal expansion of lineages carrying the mobile genetic element ICESag37 rather than being uniformly linked to disease status (Khan et al., 2024), and with genomic data from southern mainland China showing multidrug-resistance clusters within neonatal infection-associated CC17 isolates (Campisi et al., 2016). In the same context, the restricted distribution of repUS43 in Figure 3 further supports that part of the resistance background in the 104 study isolates may be linked to lineage-associated mobile genetic elements.

The virulence data suggest that the more conserved exotoxin-related genes and the more variable adhesin-associated genes play different roles in shaping the disease-associated background. In the 104 study isolates, *cfb* and the *cyl* operon were universally present, which is consistent with the view that CAMP factor and β-hemolysin/cytolysin belong to the core virulence repertoire of *S. agalactiae* rather than lineage-restricted markers (Megli et al., 2025). By contrast, the more discriminating signal came from *fbsB*, which was more frequent in neonatal infection isolates than in maternal colonizing isolates (32.7% vs. 13.5%) and, among the major clonal complexes, was restricted to CC17. This pattern is biologically plausible because *fbsB* encodes a fibrinogen-binding adhesin linked to epithelial interaction and invasion, and strains with the highest fibrinogen-binding capacity have previously been shown to belong to the highly invasive CC17 lineage (Al Safadi et al., 2011). A multicenter French genotypic study similarly identified *fbsB* as a characteristic marker of CC17 strains (Lacasse et al., 2022). In the same context, the markedly lower carriage of *hylB* in CC19 than in CC12, CC17, or CC327 further supports that exoenzyme-associated backgrounds are structured by lineage rather than being evenly distributed across neonatal infection and maternal colonizing populations.

A similarly lineage-dependent pattern was observed for pilus-associated genes. In the 104 study isolates, the PI-1-related markers and the PI-2a-related markers were common in CC19, CC12, and CC327, but were much less frequent in CC17. Published data indicate that CC/ST17 is usually not characterized by a PI-2a-associated pilus profile. In a global lineage-based study of 295 *S. agalactiae strains*, PI-2a was essentially absent from CC17, whereas PI-1 was retained in 69 of 70 CC17 strains (98.6%) (Springman et al., 2014). In a Canadian whole-genome study of 93 invasive CC17 isolates, 12 strains (12.9%) lacked PI-1, showing that PI-1 carriage can vary even within this lineage (Teatero et al., 2016). More recently, in Japanese pediatric invasive isolates, serotype III/CC17 showed PI-2b alone in 80.0% of strains and PI-1 plus PI-2b in 20.0%, while PI-2a was not detected (Kasai et al., 2026). Taken together, these data suggest that the low carriage of the PI-1/PI-2a-associated markers analyzed in the present study among CC17 isolates is compatible with the known heterogeneity of pilus gene content within this lineage, rather than being an isolated finding in the present collection.

Placing the 52 neonatal infection isolates from Southwest China into a broader public blood/CSF-derived genomic background showed that they did not form a distinct local branch, but were embedded within several established lineages. This is consistent with a genome-based study showing that human disease-associated GBS is largely structured around a limited number of successful clones rather than geographically isolated populations (Da Cunha et al., 2014). A nationwide genomic analysis of 1,345 neonatal invasive isolates from the Netherlands likewise found that most isolates clustered within five major lineages—CC17 (39%), CC19 (25%), CC23 (18%), CC10 (9%), and CC1 (7%)—and attributed the increasing disease burden to clonal expansion within existing backgrounds rather than the emergence of entirely new lineages (Jamrozy et al., 2020). Against this background, the visible aggregation of the Southwest China isolates within the CC19/III-1 and CC12/Ib clades, with additional representation in the CC17/III-2 background, suggests that neonatal GBS infection in this region may be associated with regional clustering within globally distributed lineages rather than with a uniquely local phylogenetic origin. In particular, the prominence of the CC12/Ib cluster is compatible with recent East Asian pediatric data. In Shanghai, Ib/CC12 accounted for 22.4% (13/58) of bloodstream infection isolates and ranked second only to III/CC17 (41.4%, 24/58) (Liu et al., 2021), while a Taiwan neonatal study further associated type Ib/CC12 disease with complicated sepsis in 71.4% (10/14) of cases and sepsis-attributable mortality in 42.9% (6/14) (Hsu et al., 2022).

From a clinical perspective, the findings of this study support the continued relevance of penicillin-based strategies for neonatal GBS infection in Southwest China. All 52 neonatal infection isolates remained susceptible to penicillin and ampicillin, indicating that β-lactams remain appropriate as the backbone of empirical and targeted treatment before isolate-specific susceptibility results are available. However, the high proportions of erythromycin-, clindamycin-, tetracycline-, and moxifloxacin-resistant isolates indicate that non-β-lactam alternatives should be selected cautiously. This is particularly relevant for clindamycin, which may be considered in selected β-lactam-allergic settings but should not be used without susceptibility confirmation. In addition, the observation that neonatal infection isolates were concentrated in specific serotype–CC backgrounds, especially Ib/CC12, III-2/CC17, and III-1/CC19, suggests that routine clinical microbiology should be complemented by molecular surveillance when assessing local disease burden and treatment-related risk.

These findings also have implications for maternal prevention and vaccine-oriented surveillance. Current capsular polysaccharide conjugate vaccine development has focused mainly on the serotypes responsible for most invasive infant disease. The investigational hexavalent GBS conjugate vaccine GBS6 targets serotypes Ia, Ib, II, III, IV, and V, and phase 1/2 as well as phase 2 studies have shown acceptable safety and immunogenicity profiles, including antibody responses to all six vaccine serotypes and transplacental transfer in pregnant participants or relevant clinical trial settings (Absalon et al., 2020; Smith et al., 2025). In the present study, all 52 neonatal infection isolates belonged to serotypes Ia, Ib, III, or V, which are included in the hexavalent vaccine formulation. Therefore, although this study did not evaluate vaccine effectiveness, the serotype composition of local neonatal infection isolates suggests that currently prioritized capsular vaccine strategies would theoretically cover the dominant disease-associated serotypes in this regional dataset. Continued genomic surveillance will still be necessary, because changes in clonal composition, capsular switching, or expansion of less common serotypes could alter future vaccine coverage.

This study has several strengths. First, to our knowledge, this is the first whole-genome sequencing-based study from Southwest China to evaluate neonatal GBS infection isolates together with contemporaneous maternal colonizing isolates, thereby providing a regional genomic baseline for both neonatal disease and the maternal colonizing reservoir. Second, the antimicrobial susceptibility results support that penicillin remains an appropriate first-line empirical option for suspected neonatal GBS infection before isolate-specific susceptibility results are available. Third, all neonatal infection isolates in this study belonged to serotypes Ia, Ib, III, or V, which are included in current multivalent capsular polysaccharide conjugate vaccine strategies, providing local evidence relevant to future vaccine evaluation and implementation. Finally, by placing local neonatal infection isolates within a broader public blood/CSF-derived genome background, this study offered additional phylogenetic context for interpreting the regional molecular characteristics of GBS infection in Southwest China.

Several limitations and interpretive considerations should be acknowledged. First, this was a retrospective study conducted at a single institution. However, West China Second University Hospital/National Regional Medical Center (Southwest China) is a major regional referral center for women and children with multiple campuses, and therefore the isolates may partially reflect the molecular characteristics of clinically significant neonatal GBS infections in Southwest China; nevertheless, the findings should not be interpreted as population-based incidence or prevalence estimates. Second, the maternal colonizing isolates were selected as contemporaneous population-level comparators according to predefined criteria based on sampling time and maternal age, rather than as mother–infant paired isolates. This design allowed comparison between neonatal infection isolates and the broader contemporaneous maternal colonizing population, but it was not intended to infer individual-level vertical transmission, mother–neonate strain concordance, or possible hospital environmental contamination. Third, although basic clinical outcomes were available for all neonatal cases, including clinical improvement, death, and withdrawal of treatment, detailed patient-level antimicrobial treatment regimens could not be systematically retrieved for all cases because the clinical records were distributed across independent HIS systems from different hospital campuses. Therefore, regimen-stratified analyses of treatment response or patient-level associations between antimicrobial resistance profiles and clinical outcomes could not be performed. Fourth, resistance- and virulence-related analyses were based on genomic detection of selected genes or markers. This approach was appropriate for describing lineage-associated carriage patterns, but it did not assess gene expression or experimentally determine the functional contribution of individual markers to antimicrobial resistance, colonization, or pathogenic potential.

## 5 Conclusion

This study characterized neonatal GBS infection isolates from Southwest China using phenotypic susceptibility testing and whole-genome sequencing, with parallel comparison to contemporaneous maternal colonizing isolates and a broader public blood/CSF-derived genome background. The neonatal infection isolates remained fully susceptible to penicillin and ampicillin, supporting the continued use of β-lactam-based empirical and targeted treatment, whereas resistance to erythromycin, clindamycin, tetracycline, and moxifloxacin was common. Molecular typing revealed that serotype III remained the leading serotype among neonatal infection isolates; within serotype III, III-2/CC17 was markedly enriched among neonatal infection isolates, whereas III-1/CC19 also represented an important local neonatal infection-associated background. In addition, serotype Ib and the Ib/CC12 lineage accounted for a substantial proportion of local neonatal infection isolates. Compared with maternal colonizing isolates, neonatal infection isolates were associated with selected phylogenetic backgrounds rather than directly mirroring the maternal colonizing population. Resistance- and virulence-related gene carriage was also strongly structured by clonal background, particularly among major CCs. In the global phylogenetic context, local neonatal infection isolates were embedded within established lineages represented by public blood/CSF-derived genomes, with regional clustering in selected CC-associated backgrounds. Together, these findings provide a regional genomic baseline for neonatal GBS infection in Southwest China and support continued integrated surveillance of antimicrobial susceptibility, molecular lineages, and vaccine-relevant serotypes.

## Data Availability

The two BioProject records and their associated 104 BioSample records have been released in the NCBI Submission Portal. The BioProject links are: PRJNA1449958: https://www.ncbi.nlm.nih.gov/bioproject/PRJNA1449958; PRJNA1449973: https://www.ncbi.nlm.nih.gov/bioproject/PRJNA1449973. The associated BioSample records are listed below. For PRJNA1449958, neonatal GBS infection isolates: SAMN57137507: https://www.ncbi.nlm.nih.gov/biosample/SAMN57137507; SAMN57137508: https://www.ncbi.nlm.nih.gov/biosample/SAMN57137508; SAMN57137509: https://www.ncbi.nlm.nih.gov/biosample/SAMN57137509; SAMN57137510: https://www.ncbi.nlm.nih.gov/biosample/SAMN57137510; SAMN57137511: https://www.ncbi.nlm.nih.gov/biosample/SAMN57137511; SAMN57137512: https://www.ncbi.nlm.nih.gov/biosample/SAMN57137512; SAMN57137513: https://www.ncbi.nlm.nih.gov/biosample/SAMN57137513; SAMN57137514: https://www.ncbi.nlm.nih.gov/biosample/SAMN57137514; SAMN57137515: https://www.ncbi.nlm.nih.gov/biosample/SAMN57137515; SAMN57137516: https://www.ncbi.nlm.nih.gov/biosample/SAMN57137516; SAMN57137517: https://www.ncbi.nlm.nih.gov/biosample/SAMN57137517; SAMN57137518: https://www.ncbi.nlm.nih.gov/biosample/SAMN57137518; SAMN57137519: https://www.ncbi.nlm.nih.gov/biosample/SAMN57137519; SAMN57137520: https://www.ncbi.nlm.nih.gov/biosample/SAMN57137520; SAMN57137521: https://www.ncbi.nlm.nih.gov/biosample/SAMN57137521; SAMN57137522: https://www.ncbi.nlm.nih.gov/biosample/SAMN57137522; SAMN57137523: https://www.ncbi.nlm.nih.gov/biosample/SAMN57137523; SAMN57137524: https://www.ncbi.nlm.nih.gov/biosample/SAMN57137524; SAMN57137525: https://www.ncbi.nlm.nih.gov/biosample/SAMN57137525; SAMN57137526: https://www.ncbi.nlm.nih.gov/biosample/SAMN57137526; SAMN57137527: https://www.ncbi.nlm.nih.gov/biosample/SAMN57137527; SAMN57137528: https://www.ncbi.nlm.nih.gov/biosample/SAMN57137528; SAMN57137529: https://www.ncbi.nlm.nih.gov/biosample/SAMN57137529; SAMN57137530: https://www.ncbi.nlm.nih.gov/biosample/SAMN57137530; SAMN57137531: https://www.ncbi.nlm.nih.gov/biosample/SAMN57137531; SAMN57137532: https://www.ncbi.nlm.nih.gov/biosample/SAMN57137532; SAMN57137533: https://www.ncbi.nlm.nih.gov/biosample/SAMN57137533; SAMN57137534: https://www.ncbi.nlm.nih.gov/biosample/SAMN57137534; SAMN57137535: https://www.ncbi.nlm.nih.gov/biosample/SAMN57137535; SAMN57137536: https://www.ncbi.nlm.nih.gov/biosample/SAMN57137536; SAMN57137537: https://www.ncbi.nlm.nih.gov/biosample/SAMN57137537; SAMN57137538: https://www.ncbi.nlm.nih.gov/biosample/SAMN57137538; SAMN57137539: https://www.ncbi.nlm.nih.gov/biosample/SAMN57137539; SAMN57137540: https://www.ncbi.nlm.nih.gov/biosample/SAMN57137540; SAMN57137541: https://www.ncbi.nlm.nih.gov/biosample/SAMN57137541; SAMN57137542: https://www.ncbi.nlm.nih.gov/biosample/SAMN57137542; SAMN57137543: https://www.ncbi.nlm.nih.gov/biosample/SAMN57137543; SAMN57137544: https://www.ncbi.nlm.nih.gov/biosample/SAMN57137544; SAMN57137545: https://www.ncbi.nlm.nih.gov/biosample/SAMN57137545; SAMN57137546: https://www.ncbi.nlm.nih.gov/biosample/SAMN57137546; SAMN57137547: https://www.ncbi.nlm.nih.gov/biosample/SAMN57137547; SAMN57137548: https://www.ncbi.nlm.nih.gov/biosample/SAMN57137548; SAMN57137549: https://www.ncbi.nlm.nih.gov/biosample/SAMN57137549; SAMN57137550: https://www.ncbi.nlm.nih.gov/biosample/SAMN57137550; SAMN57137551: https://www.ncbi.nlm.nih.gov/biosample/SAMN57137551; SAMN57137552: https://www.ncbi.nlm.nih.gov/biosample/SAMN57137552; SAMN57137553: https://www.ncbi.nlm.nih.gov/biosample/SAMN57137553; SAMN57137554: https://www.ncbi.nlm.nih.gov/biosample/SAMN57137554; SAMN57137555: https://www.ncbi.nlm.nih.gov/biosample/SAMN57137555; SAMN57137556: https://www.ncbi.nlm.nih.gov/biosample/SAMN57137556; SAMN57137557: https://www.ncbi.nlm.nih.gov/biosample/SAMN57137557; SAMN57137558: https://www.ncbi.nlm.nih.gov/biosample/SAMN57137558. For PRJNA1449973, maternal GBS colonizing isolates: SAMN57138562: https://www.ncbi.nlm.nih.gov/biosample/SAMN57138562; SAMN57138563: https://www.ncbi.nlm.nih.gov/biosample/SAMN57138563; SAMN57138564: https://www.ncbi.nlm.nih.gov/biosample/SAMN57138564; SAMN57138565: https://www.ncbi.nlm.nih.gov/biosample/SAMN57138565; SAMN57138566: https://www.ncbi.nlm.nih.gov/biosample/SAMN57138566; SAMN57138567: https://www.ncbi.nlm.nih.gov/biosample/SAMN57138567; SAMN57138568: https://www.ncbi.nlm.nih.gov/biosample/SAMN57138568; SAMN57138569: https://www.ncbi.nlm.nih.gov/biosample/SAMN57138569; SAMN57138570: https://www.ncbi.nlm.nih.gov/biosample/SAMN57138570; SAMN57138571: https://www.ncbi.nlm.nih.gov/biosample/SAMN57138571; SAMN57138572: https://www.ncbi.nlm.nih.gov/biosample/SAMN57138572; SAMN57138573: https://www.ncbi.nlm.nih.gov/biosample/SAMN57138573; SAMN57138574: https://www.ncbi.nlm.nih.gov/biosample/SAMN57138574; SAMN57138575: https://www.ncbi.nlm.nih.gov/biosample/SAMN57138575; SAMN57138576: https://www.ncbi.nlm.nih.gov/biosample/SAMN57138576; SAMN57138577: https://www.ncbi.nlm.nih.gov/biosample/SAMN57138577; SAMN57138578: https://www.ncbi.nlm.nih.gov/biosample/SAMN57138578; SAMN57138579: https://www.ncbi.nlm.nih.gov/biosample/SAMN57138579; SAMN57138580: https://www.ncbi.nlm.nih.gov/biosample/SAMN57138580; SAMN57138581: https://www.ncbi.nlm.nih.gov/biosample/SAMN57138581; SAMN57138582: https://www.ncbi.nlm.nih.gov/biosample/SAMN57138582; SAMN57138583: https://www.ncbi.nlm.nih.gov/biosample/SAMN57138583; SAMN57138584: https://www.ncbi.nlm.nih.gov/biosample/SAMN57138584; SAMN57138585: https://www.ncbi.nlm.nih.gov/biosample/SAMN57138585; SAMN57138586: https://www.ncbi.nlm.nih.gov/biosample/SAMN57138586; SAMN57138587: https://www.ncbi.nlm.nih.gov/biosample/SAMN57138587; SAMN57138588: https://www.ncbi.nlm.nih.gov/biosample/SAMN57138588; SAMN57138589: https://www.ncbi.nlm.nih.gov/biosample/SAMN57138589; SAMN57138590: https://www.ncbi.nlm.nih.gov/biosample/SAMN57138590; SAMN57138591: https://www.ncbi.nlm.nih.gov/biosample/SAMN57138591; SAMN57138592: https://www.ncbi.nlm.nih.gov/biosample/SAMN57138592; SAMN57138593: https://www.ncbi.nlm.nih.gov/biosample/SAMN57138593; SAMN57138594: https://www.ncbi.nlm.nih.gov/biosample/SAMN57138594; SAMN57138595: https://www.ncbi.nlm.nih.gov/biosample/SAMN57138595; SAMN57138596: https://www.ncbi.nlm.nih.gov/biosample/SAMN57138596; SAMN57138597: https://www.ncbi.nlm.nih.gov/biosample/SAMN57138597; SAMN57138598: https://www.ncbi.nlm.nih.gov/biosample/SAMN57138598; SAMN57138599: https://www.ncbi.nlm.nih.gov/biosample/SAMN57138599; SAMN57138600: https://www.ncbi.nlm.nih.gov/biosample/SAMN57138600; SAMN57138601: https://www.ncbi.nlm.nih.gov/biosample/SAMN57138601; SAMN57138602: https://www.ncbi.nlm.nih.gov/biosample/SAMN57138602; SAMN57138603: https://www.ncbi.nlm.nih.gov/biosample/SAMN57138603; SAMN57138604: https://www.ncbi.nlm.nih.gov/biosample/SAMN57138604; SAMN57138605: https://www.ncbi.nlm.nih.gov/biosample/SAMN57138605; SAMN57138606: https://www.ncbi.nlm.nih.gov/biosample/SAMN57138606; SAMN57138607: https://www.ncbi.nlm.nih.gov/biosample/SAMN57138607; SAZMN57138608: https://www.ncbi.nlm.nih.gov/biosample/SAMN57138608; SAMN57138609: https://www.ncbi.nlm.nih.gov/biosample/SAMN57138609; SAMN57138610: https://www.ncbi.nlm.nih.gov/biosample/SAMN57138610; SAMN57138611: https://www.ncbi.nlm.nih.gov/biosample/SAMN57138611; SAMN57138612: https://www.ncbi.nlm.nih.gov/biosample/SAMN57138612; SAMN57138613: https://www.ncbi.nlm.nih.gov/biosample/SAMN57138613.

## References

[B1] Absalon J. Segall N. Block S. L. Center K. J. Scully I. L. Giardina P. C. . (2020). Safety and immunogenicity of a novel hexavalent group B streptococcus conjugate vaccine in healthy, non-pregnant adults: a phase 1/2, randomised, placebo-controlled, observer-blinded, dose-escalation trial. Lancet Infect. Dis. 21, 263–274. doi: 10.1016/S1473-3099(20)30478-3 32891191 PMC9760110

[B2] Al Safadi R. Mereghetti L. Salloum M. Lartigue M.-F. Virlogeux-Payant I. Quentin R. . (2011). Two-component system RgfA/C activates the *fbsB* gene encoding major fibrinogen-binding protein in highly virulent CC17 clone group B *Streptococcus*. PLoS ONE 6:e14658. doi: 10.1371/journal.pone.0014658 21326613 PMC3033900

[B3] Bourrel A.-S. Picart A. Fernandez J.-C. Hays C. Mignon V. Saubaméa B. . (2024). Specific interaction between Group B *Streptococcus* CC17 hypervirulent clone and phagocytes. Infect. Immun. 92:e0006224. doi: 10.1128/iai.00188-24 38514466 PMC11003227

[B4] Caliot É. Dramsi S. Chapot-Chartier M.-P. Courtin P. Kulakauskas S. Péchoux C. . (2012). Role of the Group B antigen of *Streptococcus agalactiae*: a peptidoglycan-anchored polysaccharide involved in cell wall biogenesis. PLoS Pathog. 8:e1002756. doi: 10.1371/journal.ppat.1002756 22719253 PMC3375309

[B5] Campisi E. Rosini R. Ji W. Guidotti S. Rojas-López M. Geng G. . (2016). Genomic analysis reveals multi-drug resistance clusters in Group B *Streptococcus* CC17 hypervirulent isolates causing neonatal invasive disease in southern mainland China. Front. Microbiol. 7:1265. doi: 10.3389/fmicb.2016.01265 27574519 PMC4983569

[B6] Chaguza C. Jamrozy D. Bijlsma M. W. Kuijpers T. W. van de Beek D. van der Ende A. . (2022). Population genomics of Group B *Streptococcus* reveals the genetics of neonatal disease onset and meningeal invasion. Nat. Commun. 13:4215. doi: 10.1038/s41467-022-31858-4 35864107 PMC9304382

[B7] Coggins S. A. Puopolo K. M. (2024). Neonatal Group B *Streptococcus* disease. Pediatr. Rev. 45, 63–73. doi: 10.1542/pir.2023-006154 38296778 PMC10919294

[B8] Da Cunha V. Davies M. R. Douarre P.-E. Rosinski-Chupin I. Margarit I. Spinali S. . (2014). *Streptococcus agalactiae* clones infecting humans were selected and fixed through the extensive use of tetracycline. Nat. Commun. 5:4544. doi: 10.1038/ncomms7108 25088811 PMC4538795

[B9] Deshayes de Cambronne R. Fouet A. Picart A. Bourrel A.-S. Anjou C. Bouvier G. . (2021). CC17 group B *Streptococcus* exploits integrins for neonatal meningitis development. J. Clin. Invest. 131:e136737. doi: 10.1172/JCI136737 33465054 PMC7919713

[B10] Dhudasia M. B. Flannery D. D. Pfeifer M. R. Puopolo K. M. (2021). Updated guidance: prevention and management of perinatal Group B *Streptococcus* infection. Neoreviews 22, e177–e188. doi: 10.1542/neo.22-3-e177 33649090

[B11] Gonçalves B. P. Procter S. R. Paul P. Chandna J. Lewin A. Seedat F. . (2022). Group B streptococcus infection during pregnancy and infancy: estimates of regional and global burden. Lancet Glob. Health 10, e807–e819. doi: 10.1016/S2214-109X(22)00093-6 35490693 PMC9090904

[B12] Gori A. Harrison O. B. Mlia E. Nishihara Y. Chan J. M. Msefula J. . (2020). Pan-GWAS of *Streptococcus agalactiae* highlights lineage-specific genes associated with virulence and niche adaptation. mBio 11:e00728-20. doi: 10.1128/mBio.00728-20 32518186 PMC7373188

[B13] Hsu J.-F. Chen Y.-N. Chu S.-M. Lee W.-J. Huang H.-R. Chiang M.-C. . (2022). Clonal Complex 12 Serotype Ib *Streptococcus agalactiae* strain causing complicated sepsis in neonates: clinical features and genetic characteristics. Microbiol. Spectr. 11:e0377822. doi: 10.1128/spectrum.03778-22 36475780 PMC9927456

[B14] Jamrozy D. Bijlsma M. W. de Goffau M. C. van de Beek D. Kuijpers T. W. Parkhill J. . (2020). Increasing incidence of group B streptococcus neonatal infections in the Netherlands is associated with clonal expansion of CC17 and CC23. Sci. Rep. 10:9539. doi: 10.1038/s41598-020-66214-3 32533007 PMC7293262

[B15] Ji W. Liu H. Madhi S. A. Cunnington M. Zhang Z. Dangor Z. . (2019). Clinical and molecular epidemiology of invasive Group B *Streptococcus* disease among infants, China. Emerg. Infect. Dis. 25, 2021–2030. doi: 10.3201/eid2511.181647 31600132 PMC6810193

[B16] Ji W. Zhou H. Li J. Britto C. D. Liu Z. Zhang W. . (2024). Distributions of candidate vaccine targets, virulence factors, and resistance features of invasive group B *Streptococcus* using whole-genome sequencing: a multicenter, population-based surveillance study. Vaccine 42, 3564–3571. doi: 10.1016/j.vaccine.2024.04.062 38692955

[B17] Jolley K. A. Maiden M. C. J. (2010). BIGSdb: scalable analysis of bacterial genome variation at the population level. BMC Bioinformatics 11:595. doi: 10.1186/1471-2105-11-595 21143983 PMC3004885

[B18] Kasai M. Nakano S. Koide S. Otake S. Shibata M. Ishida-Kuroki K. . (2026). Prevalence of candidate vaccine targets and genomic features of pediatric invasive *Streptococcus agalactiae* in Japan. J. Infect. Dis. 233, e11–e21. doi: 10.1093/infdis/jiaf491 41065369 PMC12811887

[B19] Khan U. B. Dyster V. Chaguza C. van Sorge N. M. van de Beek D. Man W. K. . (2024). Genetic markers associated with host status and clonal expansion of Group B *Streptococcus* in the Netherlands. Front. Microbiol. 15:1410651. doi: 10.3389/fmicb.2024.1410651 39050634 PMC11266191

[B20] Lacasse M. Valentin A.-S. Corvec S. Bémer P. Jolivet-Gougeon A. Plouzeau C. . (2022). Genotypic characterization and biofilm production of Group B *Streptococcus* strains isolated from bone and joint infections. Microbiol. Spectr. 10:e0232921. doi: 10.1128/spectrum.02329-21 35357222 PMC9045227

[B21] Lamy M.-C. Dramsi S. Billoët A. Réglier-Poupet H. Tazi A. Raymond J. . (2006). Rapid detection of the highly virulent group B *Streptococcus* ST-17 clone. Microbes Infect. 8, 1714–1722. doi: 10.1016/j.micinf.2006.02.008 16822689

[B22] Le Doare K. O'Driscoll M. Turner K. Seedat F. Russell N. J. Seale A. C. . (2017). Intrapartum antibiotic chemoprophylaxis policies for the prevention of Group B streptococcal disease worldwide: systematic review. Clin. Infect. Dis. 65, S143–S151. doi: 10.1093/cid/cix654 29117324 PMC5850619

[B23] Li J. Ji W. Gao K. Zhou H. Zhang L. Mu X. . (2019). Molecular characteristics of group B *Streptococcus* isolates from infants in southern mainland China. BMC Infect. Dis. 19:812. doi: 10.1186/s12879-019-4434-0 31533652 PMC6751900

[B24] Lin F.-Y. C. Whiting A. Adderson E. Takahashi S. Dunn D. M. Weiss R. . (2006). Phylogenetic lineages of invasive and colonizing strains of serotype III group B Streptococci from neonates: a multicenter prospective study. J. Clin. Microbiol. 44, 1257–1261. doi: 10.1128/JCM.44.4.1257-1261.2006 16597848 PMC1448625

[B25] Liu J. Chen F. Guan H. Yu J. Yu J. Zhao J. . (2021). Emerging fatal Ib/CC12 hypervirulent multiresistant *Streptococcus agalactiae* in young infants with bloodstream infection in China. Front. Microbiol. 12:767803. doi: 10.3389/fmicb.2021.767803 34975795 PMC8715515

[B26] Liu L. Yan Z. He F. Chen J. Kuang L. Liu X. . (2025). Antibiotic susceptibility and molecular characterization based on whole-genome sequencing of *Staphylococcus aureus* causing invasive infection in children and women living in Southwest China during 2018-2023. BMC Microbiol. 25:47. doi: 10.1186/s12866-025-03758-2 39871143 PMC11770987

[B27] Lo C.-W. Liu H.-C. Lee C.-C. Lin C.-L. Chen C.-L. Jeng M.-J. . (2017). Serotype distribution and clinical correlation of *Streptococcus agalactiae* causing invasive disease in infants and children in Taiwan. J. Microbiol. Immunol. Infect. 52, 578–584. doi: 10.1016/j.jmii.2017.09.002 29100794

[B28] Madrid L. Seale A. C. Kohli-Lynch M. Edmond K. M. Lawn J. E. Heath P. T. . (2017). Infant Group B streptococcal disease incidence and serotypes worldwide: systematic review and meta-analyses. Clin. Infect. Dis. 65, S160–S172. doi: 10.1093/cid/cix656 29117326 PMC5850457

[B29] Manuel G. Twentyman J. Noble K. Eastman A. J. Aronoff D. M. Seepersaud R. . (2024). Group B streptococcal infections in pregnancy and early life. Clin. Microbiol. Rev. 38:e0015422. doi: 10.1128/cmr.00154-22 39584819 PMC11905376

[B30] Megli C. J. Carlin S. M. Giacobe E. J. Hillebrand G. H. Hooven T. A. (2025). Virulence and pathogenicity of group B *Streptococcus*: virulence factors and their roles in perinatal infection. Virulence 16:2451173. doi: 10.1080/21505594.2025.2451173 39844743 PMC11758947

[B31] Page A. J. Cummins C. A. Hunt M. Wong V. K. Reuter S. Holden M. T. G. . (2015). Roary: rapid large-scale prokaryote pan genome analysis. Bioinformatics 31, 3691–3693. doi: 10.1093/bioinformatics/btv421 26198102 PMC4817141

[B32] Panneflek T. J. R. Hasperhoven G. F. Chimwaza Y. Allen C. Lavin T. Te Pas A. B. . (2024). Intrapartum antibiotic prophylaxis to prevent Group B streptococcal infections in newborn infants: a systematic review and meta-analysis comparing various strategies. EClinicalMedicine 74:102748. doi: 10.1016/j.eclinm.2024.102748 39569026 PMC11577566

[B33] Raabe V. N. Shane A. L. (2019). Group B *Streptococcus* (*Streptococcus agalactiae*). *Microbiol. Spectr*. 7. doi: 10.1128/microbiolspec.GPP3-0007-2018 30900541 PMC6432937

[B34] Russell N. J. Seale A. C. O'Driscoll M. O'Sullivan C. Bianchi-Jassir F. Gonzalez-Guarin J. . (2017). Maternal colonization with Group B *Streptococcus* and serotype distribution worldwide: systematic review and meta-analyses. Clin. Infect. Dis. 65, S100–S111. doi: 10.1093/cid/cix658 29117327 PMC5848259

[B35] Schrag S. J. Zell E. R. Lynfield R. Roome A. Arnold K. E. Craig A. S. . (2002). A population-based comparison of strategies to prevent early-onset group B streptococcal disease in neonates. N. Engl. J. Med. 347, 233–239. doi: 10.1056/NEJMoa020205 12140298

[B36] Smith W. B. Seger W. Chawana R. Skogeby Z. Silmon de Monerri N. C. Feng Y. . (2025). A Phase 2b trial evaluating the safety, tolerability, and immunogenicity of a 6-valent Group B *Streptococcus* vaccine administered concomitantly with tetanus, diphtheria, and acellular pertussis vaccine in healthy nonpregnant female individuals. J. Infect. Dis. 231, e1065–e1074. doi: 10.1093/infdis/jiaf096 40036340 PMC12247796

[B37] Springman A. C. Lacher D. W. Waymire E. A. Wengert S. L. Singh P. Zadoks R. N. . (2014). Pilus distribution among lineages of group B *Streptococcus*: an evolutionary and clinical perspective. BMC Microbiol. 14:159. doi: 10.1186/1471-2180-14-159 24943359 PMC4074840

[B38] Stamatakis A. (2014). RAxML version 8: a tool for phylogenetic analysis and post-analysis of large phylogenies. Bioinformatics 30, 1312–1313. doi: 10.1093/bioinformatics/btu033 24451623 PMC3998144

[B39] Tan J. Wang Y. Gong X. Li J. Zhong W. Shan L. . (2021). Antibiotic resistance in neonates in China 2012-2019: a multicenter study. J. Microbiol. Immunol. Infect. 55, 454–462. doi: 10.1016/j.jmii.2021.05.004 34059443

[B40] Teatero S. Ramoutar E. McGeer A. Li A. Melano R. G. Wasserscheid J. . (2016). Clonal Complex 17 Group B *Streptococcus* strains causing invasive disease in neonates and adults originate from the same genetic pool. Sci. Rep. 6:20047. doi: 10.1038/srep20047 26843175 PMC4740736

[B41] Tiruvayipati S. Tang W. Y. Barkham T. M. S. Chen S. L. (2021). GBS-SBG - GBS serotyping by genome sequencing. Microb. Genom. 7:000688. doi: 10.1099/mgen.0.000688 34895403 PMC9842102

[B42] Van Dyke M. K. Phares C. R. Lynfield R. Thomas A. R. Arnold K. E. Craig A. S. . (2009). Evaluation of universal antenatal screening for group B streptococcus. N. Engl. J. Med. 360, 2626–2636. doi: 10.1056/NEJMoa0806820 19535801

[B43] Wang P. Ma Z. Tong J. Zhao R. Shi W. Yu S. . (2015). Serotype distribution, antimicrobial resistance, and molecular characterization of invasive group B *Streptococcus* isolates recovered from Chinese neonates. Int. J. Infect. Dis. 37, 115–118. doi: 10.1016/j.ijid.2015.06.019 26141418

[B44] Yan Z. Cui Y. Huang X. Lei S. Zhou W. Tong W. . (2021). Molecular characterization based on whole-genome sequencing of *Streptococcus pneumoniae* in children living in Southwest China during 2017-2019. Front. Cell. Infect. Microbiol. 11:726740. doi: 10.3389/fcimb.2021.726740 34796125 PMC8593041

[B45] Yan Z. Cui Y. Zhou W. Li W. Tan X. Chen W. . (2019). Molecular characterization of *Streptococcus pneumoniae* in children living in Southwest China and assessment of a potential protein vaccine, rPfbA. Vaccine 37, 721–731. doi: 10.1016/j.vaccine.2018.12.021 30611601

